# Addressing Nanovaccine Strategies for Tilapia

**DOI:** 10.3390/vaccines11081356

**Published:** 2023-08-11

**Authors:** Kim D. Thompson, Channarong Rodkhum, Anurak Bunnoy, Patcharapong Thangsunan, Sirikorn Kitiyodom, Pimwarang Sukkarun, Jakarwan Yostawornkul, Teerapong Yata, Nopadon Pirarat

**Affiliations:** 1 Moredun Research Institute, Pentlands Science Park, Penicuik EH26 0PZ, UK; 2 Center of Excellence in Fish Infectious (CE FID), Faculty of Veterinary Science, Chulalongkorn University, Bangkok 10330, Thailand; channarong.r@chula.ac.th (C.R.); patcharapong.t@chula.ac.th (P.T.); 3 Center of Excellence in Aquatic Animal Health Management, Department of Aquaculture, Faculty of Fisheries, Kasetsart University, Bangkok 10900, Thailand; ffisarb@ku.ac.th; 4 Wildlife, Exotic and Aquatic Animal Pathology Research Unit, Department of Pathology, Faculty of Veterinary Science, Chulalongkorn University, Bangkok 10330, Thailand; sirikorn.k@chula.acth (S.K.); jakarwan@nanotec.or.th (J.Y.); nopadon.p@chula.ac.th (N.P.); 5 Faculty of Veterinary Science, Rajamangala University of Technology Srivijaya, Nakhon Si Thammarat 90000, Thailand; pimwarang.s@rmutsv.ac.th; 6 Biochemistry Unit, Department of Physiology, Faculty of Veterinary Science, Chulalongkorn University, Bangkok 10330, Thailand; teerapong.y@chula.ac.th

**Keywords:** tilapia, *Oreochromis* sp., mucosal immunity, nanoparticles, vaccination

## Abstract

Tilapia is the world’s most extensively farmed species after carp. It is an attractive species for aquaculture as it grows quickly, reaching harvest size within six to seven months of production, and provides an important source of food and revenue for many low-income families, especially in low- to middle-income countries. The expansion of tilapia aquaculture has resulted in an intensification of farming systems, and this has been associated with increased disease outbreaks caused by various pathogens, mostly bacterial and viral agents. Vaccination is routinely used to control disease in higher-value finfish species, such as Atlantic salmon. At the same time, many tilapia farmers are often unwilling to vaccinate their fish by injection once the fish have been moved to their grow-out site. Alternative vaccination strategies are needed to help tilapia farmers accept and use vaccines. There is increasing interest in nanoparticle-based vaccines as alternative methods for delivering vaccines to fish, especially for oral and immersion administration. They can potentially improve vaccine efficacy through the controlled release of antigens, protecting antigens from premature proteolytic degradation in the gastric tract, and facilitating antigen uptake and processing by antigen-presenting cells. They can also allow targeted delivery of the vaccine at mucosal sites. This review provides a brief overview of the bacterial and viral diseases affecting tilapia aquaculture and vaccine strategies for farmed tilapia. It focuses on the use of nanovaccines to improve the acceptance and uptake of vaccines by tilapia farmers.

## 1. Introduction

Tilapia is an attractive aquaculture species because of its fast growth, reaching harvest size in six to seven months. It adapts well to its aquatic environment and is regarded as relatively disease-resistant [1]. It is the second most cultured group of finfish farmed globally after carp, with Nile tilapia (*Oreochromis niloticus*) being the most prominent tilapia species farmed. However, Mozambique tilapia (*O. masssambicus*), blue tilapia (*O. aureus*), and hybrid tilapia are also cultured [2,3,4,5]. Nile tilapia is well-suited to freshwater or low-salinity environments, while the culture of tilapia hybrids is increasing because of their ability to adapt to seawater aquaculture systems, expanding the range of tilapia aquaculture sites. For example, *O. mossambicus x O. niloticus* hybrids can tolerate a wide range of salinities [5]. Tilapia aquaculture has grown rapidly over recent decades, with an annual global production of more than 4.5 million tonnes in 2020 [6], and this is expected to increase to 7.3 million tonnes by 2030 [7,8]. Tilapia is cultured in more than 120 countries, including many low- and middle-income countries, providing an important source of food and revenue for many low-income families. While tilapia is native to Africa and the Middle East, the largest tilapia producers are in Asia. China has the largest tilapia aquaculture industry, followed by Indonesia, Egypt, Bangladesh, Vietnam, Thailand, Brazil, the Philippines, and Colombia [9].

The growth in tilapia aquaculture has seen an intensification of tilapia farming systems. This can affect the stress levels experienced by the fish in these systems. Higher stocking densities, poorer water quality, and routine fish husbandries, such as handling, transportation, and netting, are all known to induce stress [10]. This impacts the overall health of the fish and its ability to deal with environmental changes, leading to immunosuppression and compromising the fish’s natural defence mechanism against pathogens, thereby making it more susceptible to infection. Stress has been associated with increased disease episodes in tilapia aquaculture caused by various bacterial, viral, and parasitic agents [10]. Increased stocking densities can also spread pathogens within the farming system through fish-to-fish contact, promoting the emergence of new pathogens [11,12]. The trade of live fish that are sub-clinically infected with pathogens, infected fish products, or wild fish that transmit pathogens to farmed fish can also promote the spread of pathogens to unaffected farm sites and lead to new disease outbreaks [10].

## 2. Disease in Tilapia Aquaculture

The disease significantly impacts the tilapia industry because of the high levels of morbidity and mortality in diseased stock, production losses, and trade restrictions introduced in response to disease outbreaks. These issues can be economically devasting for the farmer [13]. The most predominant bacterial pathogens causing disease outbreaks in tilapia aquaculture are shown in Table 1. These include *Streptococcus agalactiae*, especially serotypes Ia, Ib, and III, *Streptococcus iniae*, *Aeromonas* spp., *Edwardsiella* spp. (*Edwardsiella tarda* and *Edwardsiella ictaluri*), *Mycobacterium marinum*, and *Francisella orientalis* [13,14,15], which were recently reclassified from *Francisella noatunensis* subsp. *orientalis* (*Fno*) [16]. Farmers in southeast Asia have seen an increase in *Flavobacterium columnare* outbreaks, often occurring concurrently with *F. orientalis* [17]. Examples of bacterial pathogens recently emerging in tilapia aquaculture include *Edwardsiella ictaluri* [18,19,20] and *Aeromonas veronii* [14,21,22,23]. The first report of *E. ictaluri* infection in farmed tilapia was described in 2012 [18]. The bacterium has subsequently spread to other geographical locations. Between 2019–2021, 26 *E. ictaluri* disease outbreaks were recorded in farmed tilapia, with accumulative mortality ranging between 30–65% [19,20]. Of the *E. ictaluri* isolates recovered, 80.8–100% were multidrug-resistant for 4–8 antimicrobials in the groups of penicillin, macrolides, sulfonamides, amphenicols, and glycopeptides [20]. *Edwardsiella anguillarum* has also recently affected tilapia in Korea [24] and South America [25]. Regarding *A. veronii*, diseases associated with motile aeromonads are often assumed to be caused by *A. hydrophila.* However, several other aeromonad species are associated with disease outbreaks in tilapia. Additionally, *A. veronii*, *A. sobri* [23,26], *A. dhakensis* [27], and *A. jandaei* [14] have all recently been identified as pathogens of tilapia.

Several viral infections have also been reported in tilapia aquaculture, caused by infectious pancreatic necrosis virus (Aquabirnavirus), nervous necrosis virus (Betanodavirus), tilapia larvae encephalitis virus (Herpesvirus), tilapia lake virus (TiLV) (Tilapinevirus), and different iridoviruses, including Bohle iridovirus (Ranavirus), infectious spleen and kidney necrosis virus (ISKNV, Megalocytivirus), and lymphocystivirus [28]. The viral pathogens that have been particularly problematic in tilapia aquaculture, especially over the past decade, are TiLV [29] and ISKNV [30,31,32], with both causing mass mortalities in farmed tilapia.

Various control measures are used to reduce the negative impact of disease outbreaks, including improving farm management and biosecurity, limiting fish movement, and administering antibiotics and other chemotherapeutics, probiotics, and functional feeds [10,33]. Without suitable control strategies, many pathogens will continue to spread within and between farming systems [10].

There is increasing concern about the use of antibiotics in aquaculture. As a result, the use of vaccines as a practical method for controlling disease in finfish aquaculture is receiving increasing attention [34]. Vaccines are now routinely used in Atlantic salmon (*Salmo salar* L.) aquaculture, and their use has been associated with reduced use of antibiotics by the Atlantic salmon aquaculture industry [35]. The uptake of vaccines for other fish species, including tilapia, has not been as positive, mainly because of a lack of commercial vaccines, poor vaccine performance, and cost. The misuse of antibiotics has been reported because of a lack of other suitable methods for controlling disease [34].

**Table 1:** Main bacterial and viral pathogens causing disease in tilapia aquaculture and the availability of commercial and/or experimental vaccines for these diseases.

Pathogen	Commercial Vaccine	Experimental Vaccine	Reference
Bacteria			
*Streptococcus agalactiae*	Yes—MSD Animal Health AQUAVAC^®^ Strep Sa *against S. agalactiae* serotype Ib, AQUAVAC^®^ Strep Sa1 *S. agalactiae* serotype Ia and Serotype III	Yes	[10]
*Streptococcus iniae*	Yes—MSD Animal Health AQUAVAC^®^ Strep Si and Pharmaq ALPHA JECT^®^ micro 1 TiLa	Yes	[10]
*Aeromonas* spp.—*A. hydrophila*; *A. veronii*, *A. sobri*, *A. dhakensis*, and *A. jandaei*.	-	Yes—for *A. hydrophila*; *A. veronii*, *A. sobri*	[14,23,26,27]
*Edwardsiella* spp.—*E. tarda*, *E. ictaluri E. Anguillarum*	-	Yes—for *E. tarda*	[18,19,20,24,25]
*Mycobacterium marinum*	-	-	[36]
*Francisella noatunensis* subsp. *orientalis* reclassified as *Francisella orientalis*	-	Yes	[15,16]
*Flavobacterium columnare*	-	Yes	[17]
**Virus**			
Infectious spleen and kidney necrosis virus (ISKNV, Megalocytivirus)	Yes—MSD Animal Health AQUAVAC^®^ IridoV	Yes	[30,31,32]
Nervous necrosis virus (Betanodavirus)	-	-	[28]
Tilapia larvae encephalitis virus (Herpesvirus)	-	-	[28]
Tilapia lake virus (TiLV) (Tilapinevirus)	-	Yes	[28,29]
Bohle iridovirus (Ranavirus)	-	-	[28]
Infectious pancreatic necrosis virus (Aquabirnavirus)	-	-	[28]
Lymphocystivirus	-	-	[28]

- No vaccines available.

## 3. Vaccine Strategies for Tilapia

Commercial vaccines are now available for a variety of fish species, with most based on formalin-killed whole-cell formulations [34]. However, a live attenuated vaccine has been licenced for use in catfish in the USA [37] and recombinant vaccines for use in Atlantic salmon [34]. Many of the vaccines available for Atlantic salmon are multivalent, contain an adjuvant, and are administered by intraperitoneal injection (IP), with some delivered as micro-dose formulations [34]. Only a few vaccines have been commercialised for tilapia. These include streptococcosis vaccines available from MSD Animal Health (AQUAVAC^®^ Strep Sa against *S. agalactiae* serotype Ib, AQUAVAC^®^ Strep *Sa1* against *S. agalactiae* serotype Ia and Serotype III, and AQUAVAC^®^ Strep Si against *S. iniae*) and from Pharmaq (ALPHA JECT^®^ micro 1 TiLa). There is also an ISKNV vaccine from MSD Animal Health (AQUAVAC^®^ IridoV). However, these vaccines are restricted to countries with regulatory approval.

There are numerous reports relating to experimental vaccines designed against various pathogens affecting tilapia. The vaccines currently under development for TiLV are very topical, mainly because of the virus’s recent, rapid spread within tilapia aquaculture [10]. These have focused on inactivated [38,39], live-attenuated [40], subunit, and DNA [41] formulations, with survival levels ranging from 58–86.7%. There are many reports of experimental vaccines for other significant tilapia pathogens that also offer good levels of protection using similar vaccine platforms as those described for the TiLV vaccines. See reviews [10,42,43,44,45] for some examples of experimental vaccines for *S. agalactiae*, *S. iniae*, *A. hydrophila*, *E. tarda*, *F. orientalis*, and *F. columnare*.

Despite vaccines being commercially available for tilapia and the vast amount of research relating to vaccine development, only 5% of tilapia are actually vaccinated [46]. The highest vaccine uptake is by tilapia farmers in Latin America (35%), while less than 1% of tilapia have been vaccinated in Asia and Africa. Vaccines are mainly administered to tilapia by IP injection, a delivery route that can provide strong, long-lasting protection. Tilapia farmers are often unwilling to vaccinate by injection once the fish have been moved for the grow-out phases on the farm. Firstly, this reluctance is due to tilapia being a cheap fish, resulting in small profit margins. Hence, vaccines need to lower production costs and reduce mortalities to justify the extra expense of using them, regardless of the vaccine’s efficacy [46]. Vaccines for tilapia need to be cheap because it is such a low-value species; otherwise, the farmer is unwilling to pay for the vaccine [17]. Secondly, since most vaccines are administered by injection, it is logistically challenging to vaccinate fish once they are in their grow-out site. It takes human resources, time, and investment, which many small-scale tilapia farmers do not have [46]. It is also stressful for the fish, which can exacerbate disease issues. Also, only healthy fish should be vaccinated for an optimal immune response to the vaccine. The ideal solution is to vaccinate fish in the hatchery before moving them to their grow-out site around one-month post-hatch. However, it is tricky to vaccinate small fish by injection, and costs can also be a constraint for vaccinating fish in hatcheries prior to deployment.

Immersion vaccination is widely used to vaccinate small fish, while the cost of mass vaccinating larger fish via this route is prohibitive for the tilapia farmer because of the amount of vaccine that would be required. The efficacy of immersion vaccines tends to be lower than that of IP-administered vaccines because of poor antigen uptake through the mucosa-associated lymphoid tissue (MALT), including skin, gills, nasopharynx, and lateral line pores. They often result in a shorter duration of protection [44]. Antigens must cross the mucosal barriers to be taken up by antigen-presenting cells (APCs). The APCs present antigens to the adaptive immune system, stimulating an adaptive memory response to the vaccine [47]. Factors such as the concentration and physical nature of the antigen used, the immersion time, the size of the fish, stress, pH, the salt concentration of the vaccine, and water temperature can influence antigen uptake and response to immersion vaccination [48].

Vaccines delivered orally through the tilapia’s diet would be one of the farmer’s methods for vaccinating fish [46]. It is easier to mass vaccinate fish with this method, and the costs associated with vaccination are significantly reduced. This route of administration also improves fish welfare by removing vaccination-related stress. However, the poor efficacy associated with oral formulations has prevented oral vaccines from being fully exploited [47]. The limited effectiveness of oral vaccines has been associated with the breakdown of antigens by the harsh conditions within the gastric tract and the development of tolerance to the antigen [49]. In addition, the dose of vaccine individual fish receives can be variable. This may affect the level of immunisation between individuals and their level of protection.

Most commercial tilapia injection vaccines are adjuvanted to improve the fish’s response to the vaccine [50] and to activate specific T and B lymphocyte responses [51]. Adjuvants are classified as Signal 1 (promoting antigen presentation) or Signal 2 (providing secondary co-stimulatory signals during antigen recognition signals) facilitators [50]. Commercial adjuvants such as Montanides from Seppic SA are widely used in injection vaccines for fish, while limited adjuvants are available for mucosal vaccination (immersion and oral vaccines) [34,52,53,54].

Because skin, gills, and gut are important routes for pathogen entry in teleosts, immersion and oral vaccines are more appropriate routes of vaccination from the viewpoint of stimulating mucosal immunity. The thick mucus layer covering mucosal tissues of teleosts is rich in biologically active molecules with biostatic and biocidal activities (e.g., agglutinins, antimicrobial peptides, antibodies, complement, C-reactive proteins, haemolysins, lectins, lysozymes, proteases, and proteolytic enzymes). This helps prevent the entry and proliferation of bacteria and viruses. Mucosa-associated lymphoid tissues (MALT) provide an important first-line defence against invading pathogens. These are associated with skin [skin-associated lymphoid tissue (SALT)], gut [gut-associated lymphoid tissue (GALT)], gills [gill-associated lymphoid tissues (GIALT)], nares [nasopharynx-associated lymphoid tissue (NALT)], and buccal mucosa and pharyngeal mucosa [55,56,57,58,59,60,61]. Leukocytes (lymphocytes, macrophages, and granulocytes, including eosinophilic granular cells), present in the MALT, provide innate or adaptive immune responses at a mucosal level. B- and T-cells are diffusely scattered throughout these tissues (D-MALT) and respond to both mucosal infection and vaccination (GALT [62,63,64]; SALT [65], NALT [66,67], and GIALT [68]). The interbranchial lymphoid tissue (ILT), first described in Atlantic salmon, is located at the base of the gill filaments and contains a more structured distribution of immune cells referred to as organised MALT [69]. While the role of MALTs in tilapia mucosal immunity has yet to be fully elucidated, the presence of an ILT-like structure has been reported [70].

The tilapia aquaculture industry clearly needs alternative vaccine delivery methods that are cheap, safe, and easy to administer. The use of nanoparticle vaccine delivery systems applied orally or by immersion may be one solution for promoting the use of vaccines in the tilapia aquaculture sector.

## 4. Nanoparticles as Vaccines

Nanoparticle-based formulations offer several advantages for improving vaccine design for tilapia compared with conventional formalin-killed vaccines [71,72,73,74]. One of the key advantages of nanovaccines is their ability to deliver antigens directly to the target cells of the immune system, thereby enhancing immune responses. This targeted delivery can lead to a more robust and specific immune response, resulting in increased vaccine efficacy. Antigens are either encapsulated within nanoparticles or displayed on their surface. Antigens encapsulated within the nanoparticle are protected from degradation by the harsh conditions of the fish’s gastric tract, making them attractive candidates for oral delivery to fish. Antigens on the surface of the particle facilitate interaction with pattern recognition receptors (PRRs), such as toll-like receptors (TLR), on the surface of APCs, promoting antigen uptake by the APCs [74]. Compared with unconjugated antigens, this uptake stimulates robust innate, humoral, cellular, and mucosal immune responses [73]. The adjuvating properties of nanoparticles enhance the immunogenicity of weakly immunogenic proteins, such as recombinant proteins [75]. The large surface area of the particles enables higher antigenic loads to be incorporated compared to conventional vaccines [75]. Nanoparticles can increase the solubility and permeability of the vaccine, produce increased mucosal immunity, have fewer side effects than injection vaccines, and provide targeted delivery of the vaccine to the mucosal tissues [76,77]. Positively charged particles tend to be internalised by immune cells at the site of vaccine delivery, resulting in enhanced immune responses with these particles [78,79,80]. Prolonged antigen release from the particle also reduces the need for booster vaccinations [76,77]. Additionally, nanovaccines can be designed to have sustained release properties, allowing for prolonged antigen presentation and immune stimulation, which can further enhance the vaccine’s effectiveness.

The size of the particle is related to its ability to induce an effective immune response, with smaller particles exerting a better immune response than larger particles [81]. Antigen retention by dendritic cells is also affected by nanoparticle size. Larger nanoparticles (50–100 nm) are retained for over five weeks and elicit a 5-fold greater immune response compared with smaller particles (5–15 nm), which are only retained for about 48 h [82], and this provides the immune system with a long duration of exposure to the antigen.

**Figure 1:**
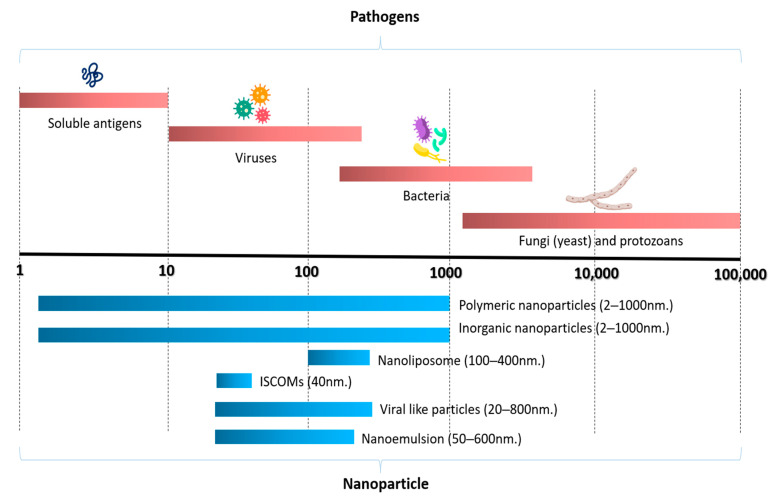
The size of nanoparticles used in fish vaccines relative to the size of pathogens (modified from [71,87]). Reprinted/adapted with permission from Ref. [87]. Copyright 2010, Springer Nature, License Number 5603701492972.

A variety of nanoparticle formulations of varying sizes have been used as vaccine platforms (Figure 1). These include inorganic and polymeric nanoparticles, nanoliposomes, nanoemulsions, immunostimulating complexes (ISCOMs), virus-like particles (VLPs), and nanotubes. Further information about the nanoparticles detailed below can be found in the following articles [34,71,72,73,74,75,83].

Inorganic nanoparticles, including gold, carbon, calcium phosphate, nickel, cobalt, and quantum dots, are nanoscale particles composed of non-organic materials. These nanoparticles possess excellent physicochemical properties for vaccine formulation, such as a high surface area-to-volume ratio, enabling efficient antigen adsorption and modification to enhance stability and controlled release of antigens. Due to their versatility, they are well-suited for delivering a wide range of fish vaccine antigens. Additionally, inorganic nanoparticles can serve as adjuvants, bolstering the immune response and significantly improving the efficacy of fish vaccines against various fish pathogens [71,72].

Polymeric nanoparticles, consisting of biodegradable and biocompatible polymers, provide a versatile platform for encapsulating fish vaccine antigens. Their nanoscale size and tailored surface properties allow prolonged antigen retention and protection against degradation. Polymeric nanoparticles can enhance antigen uptake by antigen-presenting cells and promote immune responses in fish by controlling the polymer composition, size, and surface charge. Their sustained antigen release supports long-lasting protection, reducing the need for frequent booster vaccinations. Examples of natural polymeric nanoparticles include chitosan, hyaluronic acid, and alginate. Chitosan has been widely used as a biodegradable polymeric nanoparticle that has been shown to enhance mucosal immunity in orally vaccinated fish [71]. Synthetically derived polymers such as poly lactic-co-glycolic acid (PLGA) and poly-lactic acid (PLA) have also been used to deliver peptides, synthetic proteins, and nucleic acids in human vaccines [71,84]. Moreover, these polymers have been tested as oral vaccines in fish [84,85,86].

The use of nanoliposomes in fish vaccines offers a range of advantages that contribute to their effectiveness. These lipid-based nanoparticles have a unique structure with a hydrophilic core and hydrophobic outer layers, enabling them to efficiently encapsulate hydrophilic and hydrophobic antigens. This encapsulation ensures the antigens remain protected from enzymatic degradation during vaccine delivery, leading to a more potent immune response. Additionally, nanoliposomes can be engineered to target specific immune cells, facilitating targeted antigen presentation and maximising the activation of the immune system [71,72]. Their biocompatibility ensures safe interactions with fish immune cells and tissues, making them a safer choice for vaccine delivery.

Nanoemulsions are stable, nanoscale emulsions composed of oil and water phases stabilised by surfactants. Their droplet sizes are in the nanometre range, and they are highly efficient in encapsulating different antigens. For example, hydrophobic antigens can be efficiently incorporated into the oil phase, while hydrophilic antigens can be encapsulated in the water phase. This versatility allows for improved solubility and stability of the antigens, ensuring their integrity during storage and vaccine delivery. Due to their small droplet size, nanoemulsions facilitate the controlled and sustained release of antigens. This prolonged antigen exposure leads to extended immune stimulation in fish, which is essential for developing a robust and long-lasting immune response against pathogens [71,72].

ISCOMs are self-assembling cage-like structures that consist of saponins, cholesterol, phospholipids, and antigens. These nanoparticles efficiently deliver antigens to immune cells, resulting in enhanced immunogenicity. ISCOM-based vaccines can induce both humoral and cellular immune responses, providing strong protection against various fish pathogens [71,72].

Carbon nanotubes (CNT) have several qualities that make them a good option for vaccine formulations. They serve as a scaffold for the antigenic target, enhancing its presentation to the fish’s immune system. Additionally, CNTs are inert, non-immunogenic, and non-toxic. Their unique structure enables the simultaneous attachment of various antigens to their surfaces. Moreover, CNTs can efficiently enter cells, including dendritic cells, which is crucial for provoking a robust and efficient immune response [71,72].

VLPs are self-assembled nanoparticles that bear a resemblance to viruses in structure but do not contain viral genetic material. They are designed to display specific antigens on their surface, effectively mimicking natural infections and provoking a strong immune response without causing actual disease. VLP-based fish vaccines have demonstrated efficacy against various fish pathogens, making them promising candidates for broad-spectrum protection. These engineered VLPs efficiently present antigens on their surface, triggering robust immune responses in vaccinated fish. Due to their safety and immunogenicity, VLPs hold significant potential for developing effective fish vaccines [34,71,72].

Live-attenuated vaccines tend to provide long-lasting immunity. They contain pathogen-associated molecular patterns (PAMPs), which are recognised by PRRs on host immune cells, such as TLRs. The ability of live attenuated vaccines to stimulate the host’s immune response means that adjuvants are not generally needed in the vaccine. Biologically derived nanoparticles, such as virus-like particles, outer membrane vesicles, and protein nanocages, may be a safe alternative to these since they are unable to replicate and are not infectious. They mimic the structure and function of live pathogens and contain PAMPs, thus removing the requirement for an adjuvant. There are now four FDA-approved VLP vaccines and two FDA-approved OMV vaccines for humans [74].

An overview of the advantages and disadvantages of nanovaccines for tilapia is presented in Table 2.

**Table 2:** Advantages and Disadvantages of Nanovaccines for Tilapia.

Advantages	Disadvantages
Enhanced Immune Response: Nanovaccines can improve the immune response of tilapia due to their ability to deliver antigens in a targeted and efficient manner. This could lead to better protection against pathogens.	Research and Development Challenges: Developing effective nanovaccines requires complex research and specialised knowledge. It may take time and resources to optimise formulations specific to tilapia.
Reduced Dosage: Nanovaccines may require smaller vaccine doses due to their increased potency and targeted delivery. This can reduce the overall vaccine cost and minimise the potential for environmental impact from the excess vaccine.	Regulatory Hurdles: Novel vaccine technologies like nanovaccines may face regulatory scrutiny, leading to delays in approval and commercialisation.
Controlled Release: Nanovaccines can be designed to release antigens slowly over time, ensuring a more sustained immune response and potentially longer-lasting protection.	Cost: Nanovaccines might initially be more expensive to produce than traditional vaccines, potentially limiting their widespread adoption, especially in developing regions, but costs should decrease as new processing technology is adopted.
Better Stability: Nanoparticles can protect vaccine antigens from degradation, improving the stability and shelf life of the vaccine, which is especially beneficial in aquaculture settings.	Safety Concerns: While nanomaterials are generally considered safe, there may be potential concerns regarding nanoparticle toxicity or unintended environmental effects if nanoparticles are not adequately studied.
Less Adjuvant: Traditional vaccines often require adjuvants to boost the immune response. Nanovaccines might need fewer adjuvants or have built-in adjuvant properties, reducing the risk of adverse reactions.	Limited Knowledge: The use of nanovaccines in aquaculture is still an emerging field, and there might be uncertainties related to their long-term effects on fish health and the environment
Enhanced Storage and Transport: Nanovaccines’ improved stability can facilitate easier storage and transportation, making them more accessible and suitable for remote or challenging aquaculture locations.	Technological Complexity: The development and production of nanovaccines require specialised expertise and technology, which may limit their availability in some regions

## 5. Experimental Nanovaccines for Tilapia

There are relatively few nanoparticle vaccine studies reported on tilapia. These include immersion vaccines against *F. columnare* [70,88,89,90], *A. veronii* [91], TiLV [92], and *F*. *orientalis* [90,93], oral vaccines for *S. agalactiae* [94,95,96] and *F. columnare* [96], DNA vaccines for TiLV [97,98], and a β-galactosidase reporter gene [99]. An overview of studies using nanoparticles as vaccines for tilapia is presented in Table 3.

**Figure 2:**
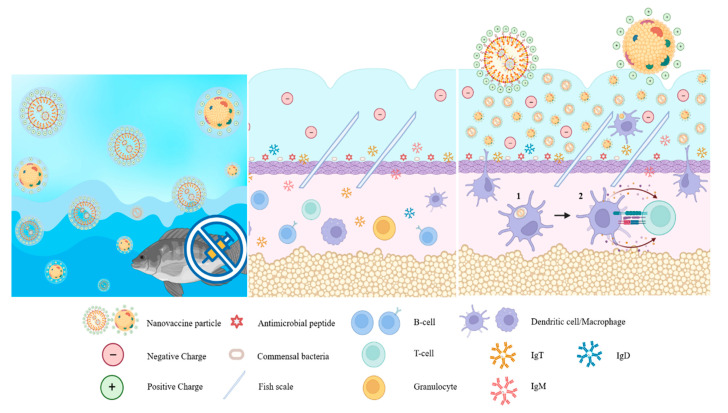
Nanoparticles facilitate immune modulation of antigen-presenting cells to promote: (1) phagocytosis of the pathogen by macrophages; (2) which then presents the antigen to CD4+ T cells through major histocompatibility complex (MHC) class II as indicated by the arrows.

Kitiyodom et al. developed an experimental immersion vaccine against *F. columnare*, the causative agent of columnaris disease, using a chitosan-coated mucoadhesive nanovaccine (CS-NE), which they tested in red tilapia [70,88,89]. Columnaris disease is an important bacterial disease of tilapia, especially during the fry and fingerling stages of production. It causes lesions on the mucosal surface of fish, particularly their skin and gills [88]. The mucoadhesive chitosan biopolymer coating gives the particles a positive charge and increases their size. The positive charge enhances the ability of the nanoparticle to attach to the surface of the gills compared to a nonencapsulated vaccine. The authors suggest that the chitosan biopolymer gives the nanoparticles “pathogen-like” properties, mimicking the mucoadhesive characteristic of live *F. columnare* [70,88]. The presence of a positive charge on nanovaccine particles improves their capacity to adhere to the mucosal surface by utilising the electrostatic mechanism. This attribute grants the nanoparticles similar pathogen characteristics, effectively mimicking their mucoadhesive properties (Figure 2). The CS-NE vaccinated group exhibited a 78% relative percentage survival (RPS) following experimental infection with Flavobacterium columnare at 30 days post-vaccination (dpv). The histological examination of the mucosal-associated lymphoid tissue (MALT) revealed notably elevated levels of leucocytes and antigen uptake in the CS-NE vaccinated fish, in contrast to both the control group and those vaccinated with whole-cell vaccines. Moreover, a significant up-regulation of key genes such as IgT, IgM, TNF-α, IL1-β, and MHC-1 was observed in the gill tissue of the CS-NE vaccinated group [70]. Besides effectively stimulating a robust mucosal immune response against columnaris disease, the CS-NE vaccine demonstrated a strong humoral systemic immune response [89]. Specific anti-antibody responses were significantly higher in the CS-NE-vaccinated fish than those vaccinated with the formalin-killed vaccine or control fish at 14 and 21 dpv. In CS-NE-vaccinated fish, there was a notable and statistically significant increase in the expression of IgM and IgT genes in the spleen.

The same nanoparticle formulation described above was used to immersion vaccinate red tilapia against *A. veronii*. This had increased efficacy compared to the control group of fish vaccinated with an empty-polymeric nanovaccine and a formalin-killed bacterin vaccine when measured at 30 dpv [91]. However, the authors had concerns about the stability and reproducibility of the chitosan-based platform [101]. Therefore, they assessed cetyltrimethylammonium bromide (CTAB), a cationic surfactant, as a mucoadhesive nanovaccine platform to prepare a cationic *Fno* nanovaccine (CAT-Fno-NV) for immersion vaccination of red tilapia against *F. orientalis* [93]. The CAT-Fno-NV vaccinated group had the highest level of protection against an experimental *F. orientalis* infection, based on the bacterial load in the head kidney, spleen, and liver of CAT-Fno-NV vaccinated fish at 30 days post-challenge. Significant upregulation of IgM transcripts was seen in the gills, skin, head kidney, serum, peripheral blood lymphocytes, and spleen tissues of fish vaccinated with whole cells or the CAT-Fno-NV vaccine. In contrast, a significant increase in IgT transcripts was only seen in the gills and skin of vaccinated fish.

The efficacy of a chitosan nanoparticle TiLV immersion vaccine (CN-KV) was tested in the laboratory using a cohabitation model and in field trials [92], with RPS levels of 68.17% and 52.2%, respectively [92].

A novel oral delivery system based on nanoclay, halloysite nanotubes (HNTs), and modified forms of these [HNT-Chitosan (HC), HNT-APTES (HA), and HNT-APTES-Chitosan (HAC)] was evaluated as a nanovaccine against streptococcosis in tilapia (*Oreochromis* sp.). The nanotubes were filled with killed *S. agalactiae* (serotypes Ia and III) and fed to tilapia for seven days in weeks one and three of the trial [94]. The efficacy of the vaccine was based on specific antibody levels in vaccinated fish and protection against an experimental infection with the two *S. agalactiae* serotypes. The highest specific antibody level was against *S. agalactiae* serotype Ia in HCF orally administered fish. This group had a significant RPS value of 75.0 ± 10.8% when experimentally infected with serotype III.

Another oral vaccine against streptococcosis in tilapia has been trialled using a poly [(methyl methacrylate)-co-(methyl acrylate)-co-(methacrylic acid)]-poly(d,l-lactide-co-glycolide) (PMMMA-PLGA) particle [95]. The surface immunogenic protein (SIP) of *S. agalactiae*, produced as a recombinant protein, was encapsulated in the nanoparticles and administered orally to tilapia three times, with a 7-day interval between each immunisation. The SIP antigen was localised in the colon, spleen, and kidney of vaccinated fish; SIP-specific antibodies were detected in the orally vaccinated fish; and 100% of the orally vaccinated tilapia were protected from *S. agalactiae* infection. The authors suggest that the negative charge of the particles, produced by ionisation of the carboxyl groups in PMMMA, shielded the nanoparticles from uptake by small intestinal epithelial cells.

A cationic-based nanoemulsion containing bile salts and coated with chitosan (NEB-CS) has also given promising results when used as an oral vaccine against *S. agalactiae.* The vaccine antigen was protected inside the core of the encapsulated bile salt nanocarrier, providing higher stability in the gastrointestinal tract. Incorporating NEB-CS into the feed significantly enhanced the vaccine’s mucoadhesiveness, permeability, and overall protective efficacy. This promising approach indicates that NEB-CS has the potential to effectively safeguard tilapia in aquaculture against streptococcosis [96].

In the study by Leal et al. [100], alginate microparticles containing *F. columnare* were administered orally to Nile tilapia and did not stimulate an antibody response in the fish. In contrast, IP and intramuscular (IM) administration did elicit a response. However, the antibody response of vaccinated fish did not reflect any resistance to the pathogen when vaccinated fish were challenged with the pathogen.

Regarding DNA vaccines, these are usually administered by IM injection. In the study by Ramos et al. [99], a DNA construct expressing β-galactosidase as a reporter gene was delivered orally to fish, with the construct encapsulated in chitosan. β-galactosidase expression was observed in the fish’s stomach, spleen, and gills. The authors suggested that encapsulated DNA constructs could be an easy and cheap way of delivering DNA vaccines to tilapia through their diet. However, they did not test any vaccine for efficacy using the nanoparticle.

A biomimetic nanodelivery system (Cs-pS2@M-M) using a mannose-modified erythrocyte membrane was used as a vaccine carrier for a DNA vaccine against TiLV [97]. This was injected IM into tilapia, and its efficacy was evaluated based on specific antibody responses, immune gene expression, and RPS in a TiLV challenge. The Cs-pS2@M-M nanoparticles provided a 76.9% RPS, which was 26.9% higher than a naked DNA vaccine (pS2) and 15.4% higher than that of Cs-pS2@M without mannose modification.

## 6. Conclusions and Future Direction

In the absence of practical ways for the tilapia industry to manage disease issues, vaccination offers a realistic approach to help with this. Vaccination is a valuable component of fish health management, contributing to healthier fish, enhanced growth, and improved quality in aquaculture operations. Vaccination plays a crucial role in aquaculture by preventing and controlling infectious diseases in farmed fish. By reducing disease incidence and mortality rates, vaccinated fish experience less illness-related stress, positively impacting their growth and overall quality. Effective vaccination programmes lead to improved feed conversion, allowing fish to efficiently convert feed into body mass and achieve better growth rates. Advancements in vaccine delivery methods, such as nanovaccines, offer less invasive and stressful alternatives for fish during the vaccination process. The use of vaccines would allow the tilapia industry to grow sustainably and safely, but the uptake of vaccines by tilapia farmers needs to be improved. Tilapia farmers are often reluctant to administer vaccines by injection once the fish have been transferred to their grow-out phase. The main reasons behind this reluctance are the economic aspect—tilapia being a low-cost product with small profit margins—and the logistical difficulty of vaccinating fish once they have been moved. Consequently, farmers demand vaccines that can demonstrate tangible benefits, such as reduced production costs and lower mortality rates, to justify the added expense of vaccination. Vaccinating fingerlings in the hatchery by immersion vaccination and mass vaccination through oral delivery once fish are in their grow-out site should make vaccination for tilapia farmers easier and cheaper. Nanoparticles present a promising opportunity to enhance the effectiveness of immersion and oral vaccines, enabling large-scale vaccination of fish through these routes. To develop vaccines that offer optimal protection, it is essential to gain a deeper understanding of the mucosal immune responses of tilapia to various nanoparticles. Additionally, confirming the stability and safety of these nanoparticles is crucial. The prevalence of concurrent infections in tilapia cultures adds complexity to vaccine design, potentially necessitating the use of multivalent vaccine platforms to address these challenges. Another advantage of nanovaccines is their potential for combination or multivalent vaccines. By incorporating multiple antigens into a single nanovaccine formulation, it becomes possible to protect against multiple pathogens simultaneously, reducing the number of vaccine administrations and simplifying vaccination protocols. This can greatly improve the efficiency and cost-effectiveness of vaccination programmes in tilapia aquaculture.

Nanovaccines offer enhanced stability and protection of antigens during storage and transportation, reducing the risk of vaccine degradation and ensuring their potency. This is especially important in tilapia aquaculture, where vaccines may need to be transported to remote locations or stored in suboptimal conditions.

Despite the considerable potential, there are still obstacles to overcome in developing and implementing nanovaccines for tilapia. These obstacles include scalability, cost-effectiveness, regulatory approval, and public acceptance. However, ongoing research and technological advancements suggest that these challenges can be addressed in the future. Priority should be given to the transfer of innovative processes from laboratory-based nanovaccine production to industrial-scale production. Upscaling fish nanovaccine production presents several challenges, including the need for more complex manufacturing facilities compared to laboratory settings and stricter industrial BSL (biosafety level) standards. These challenges can impact the management costs of vaccine production. The challenge will be to develop nanovaccines that are economically viable for the tilapia farmer to use.

## Figures and Tables

**Table 3:** Experimental nanovaccines for tilapia.

Pathogen	Nanoparticle	Route of Delivery	Relative Percentage Survival (%)	Reference
*Flavobacterium columnare*	Chitosan-coated mucoadhesive	Immersion	78%, 85%, and 72%, respectively	[70,88,89]
*Flavobacterium columnare*	Alginate	Oral	No difference between vaccinated and unvaccinated fish	[100]
*Aeromonas veronii*	Chitosan-coated mucoadhesive nanovaccine	Immersion	75%	[91]
*Francisella orientalis*	Cetyltrimethylammonium bromide	Immersion	Not determined	[93]
*Francisella orientalis* (*Fo*) and/or *Flavobacterium columnare* (*For*)	Cetyltrimethylammonium bromide	Immersion	Fish vaccinated with Fo, For, or bivalent nanovaccine and challenged with Fo were 62.5%, 6.25%, and 25%, respectively. When these fish were challenged with For, RPS values were 5.56%, 50%, and 38.9% for the Fo, For, and bivalent mucoadhesive nanovaccines groups, respectively. At the same time, co-infection with mixed antigens (Fo and For) produced RPS values of 20%, 25%, and 55% for the Fo, For, and bivalent mucoadhesive nanovaccine groups, respectively.	[90]
*Streptococcus agalactiae*	Nano clay, halloysite nanotubes (HNTs) HNT-Chitosan; HNT-APTES; and HNT-APTES-Chitosan	Oral	RPS of 75.0 ±10.8% when experimentally infected with serotype III	[94]
*Streptococcus agalactiae*	Poly [(methyl methacrylate)-co-(methyl acrylate)-co-(methacrylic acid)]-poly(d,l-lactide-co-glycolide) (PMMMA-PLGA)	Oral	100%	[95]
*Streptococcus agalactiae*	Cationic-based nanoemulsion containing bile salts and coated by chitosan	Oral	96% with homologous *S. agalactiae* Ia challenge	[96]
Tilapia lake virus	Biomimetic nano delivery system (Cs-pS2@M-M) for DNA construct using a mannose-modified erythrocyte membrane	Intramuscular	76.0% and 69.9%, respectively	[97,98]
Tilapia lake virus	Chitosan-coated mucoadhesive	Immersion	RPS of 68.17% with cohabitation challenge. Under the field trial, an RPS of 52.2% was obtained with chitosan-nanovaccine.	[99]
β-galactosidase reporter gene	DNA construct encapsulated in chitosan	Oral, intrabuccal or intramuscular		[100]

## Data Availability

No data were used for the research described in the article.
